# List of Reviewers

**DOI:** 10.1002/pcn5.70238

**Published:** 2025-11-11

The Editorial Board members of Psychiatry and Clinical Neurosciences Reports are grateful to the following reviewers who provided the Journal with their expertise from 1st October 2024 to 30th September 2025. We are also pleased to inform you that, thanks to your invaluable contributions, our journal has been granted an Impact Factor this year. Their efforts are greatly appreciated.

Abe, Takaaki

Abiko, Yoshihiro

Adachi, Hiroyoshi

Adachi, Naoto

Akizuki, Nobuya

Alag, Poorvanshi

Alpers, Silvia Eiken

Amano, Mizuki

Amemori, Kenichi

Aoki, Nobuatsu

Aoki, Yumi

Aoki, Yuta

Aoyama, Shinsuke

Arai, Yu

Arslan, Mustafa

Asada, Takahiro

Azizan, Azliyana

Baba, Hajime

Bartova, Lucie

Boku, Shuken

Brown, Ryan

Burns, Leonard

Chen, Chong

Chiba, Toshinori

Chigusa, Yoshitsugu

Choi, Tae Young

Coppola, Giangennaro

Das, Soumitra

Doi, Mana

Ercoli, Linda

Eto, Nobuaki

Ewais, Tatjana

Faradz, Sultana M. H.

Ferentinos, Panagiotis

Fujii, Rintaro

Fujishiro, Hiroshige

Fujita, Junichi

Fujiwara, Masaki

Fukui, Eriko

Fukumoto, Kentaro

Funatogawa, Tomoyuki

Funayama, Michitaka

Furihata, Ryuji

Futenma, Kunihiro

Gandy, Milena

Gidisso, Gizachew

Harima, Katsuki

Hasanvandi, Saba

Hasegawa, Naoya

Hashimoto, Naoki

Hashimoto, Satoshi

Hata, Masahiro

Hatta, Kotaro

Hayasaki, Gaku

Hayashi, Wakaho

Hicks, Amelia J.

Higuchi, Fumihiro

Hikichi, Hiroyuki

Hirakawa, Hirofumi

Hirano, Jinichi

Hirano, Yoji

Hirata, Takashi

Hirota, Tomoya

Horai, Tadasu

Hori, Arinobu

Hori, Hikaru

Horinouchi, Toru

Huertas, Rafael

Hustle, Joe

Iacono, Diego

Iga, Junichi

Ihara, Sakae

Iida, Hitoshi

Iida, Naoko

Ikebuchi, Emi

Ikeda, Akifumi

Ikeda, Eiji

Ikeda, Nozomu

Ikeda, Shunichiro

Ikenouchi, Atsuko

Imai, Atsushi

Imai, Hissei

Inaba, Hiromichi

Inoue, Keisuke

Inoue, Takeshi

Ishikawa, Shinichi

Ishizuka, Kanako

Isobe, Masanori

Ito, Masanobu

Iwaki, Hirotaka

Iwamoto, Kunihiro

Iwasa, Hajime

Iwata, Masaaki

Iwata, Yusuke

Jinno, Atsushi

Jitoku, Daisuke

Kageyama, Takayuki

Kaji, Namiko

Kanata, Sho

Kanazawa, Tetsufumi

Kanehara, Akiko

Kano, Yukiko

Katayama, Munenori

Katayama, Nariko

Kato, Hidekazu

Katsuragawa, Shuichi

Kawabe, Kentaro

Kawai, Keisuke

Kawakatsu, Shinobu

Keles Altun, İlkay

Kihara, Hiroaki

Kikuchi, Saya

Kikuchi, Yuki

Kishi, Taro

Kishi, Yasuhiro

Kobayashi, Ryota

Kobayashi, Toshiyuki

Koda, Masahide

Kodaka, Fumitoshi

Koga, Minori

Koga, Yoshiki

Koh, Joyce Suang Bee

Koizumi, Teruki

Kono, Toshiki

Kosaka, Hirotaka

Koshiyama, Daisuke

Kozuki, Haruka

Kubota, Manabu

Kuga, Hironori

Kuge, Rie

Kumazaki, Tsutomu

Kuramochi, Izumi

Kuroki, Toshihide

Kurose, Shin

Kyan, Ryoko

Lane, Hsien‐Yuan

Lin, Chung‐Ying

Liu, Jenny

Loch, Alexandre Andrade

Mariano, Marika

Maroney, Megan

Marutani, Toshiyuki

Mashimoto, Masaya

Matsubara, Tomoyasu

Matsubara, Toshio

Matsuda, Osamu

Matsuda, Yuki

Matsui, Kentaro

Matsumoto, Junya

Matsumoto, Kazuki

Matsumoto, Takuya

Matsumoto, Toshihiko

Matsuoka, Teruyuki

Matsushima, Toshio

Mei, Wei

Metoki, Hirohito

Mikami, Katsunaka

Minami, Shota

Mishima, Ryo

Mitsuboshi, Satoru

Miura, Itaru

Miyajima, Miho

Miyake, Nobumi

Miyawaki, Dai

Mizuno, Eriko

Mizuno, Satomi

Mizuno, Yuya

Momota, Yuki

Monden, Yukifumi

Morimoto, Takafumi

Morishima, Ryo

Mukai, Keiichiro

Murayama, Keitaro

Nagamine, Masanori

Nagamine, Takahiro

Nakagami, Yukako

Nakajima, Shinichiro

Nakamura, Dan

Nakamura, Mitsuru

Nakanishi, Daisuke

Nakanishi, Miharu

Nakataki, Masahito

Nakatani, Eiji

Nakazato, Michiko

Narishige, Ryuichiro

Narita, Hisashi

Narita, Kenji

Narita, Zui

Narumoto, Jin

Nasa, Prashant

Nemoto, Kiyotaka

Nemoto, Takahiro

Niimura, Hidehito

Nishida, Keiichiro

Nishida, Masaki

Nishikawa, Naoto

Nishizono‐Maher, Aya

Noda, Masataka

Noda, Sachiko

Noda, Shota

Noda, Yoshihiro

Nogimura, Akane

Nouri, Hosein

Nshida, Takuji

Numata, Shusuke

Ochi, Shinichiro

Ogata, Haruhiko

Ogawa, Asao

Ogawa, Mariko

Oguchi, Yoshiyo

Ohashi, Kei

Ohyama, Kakusho

Ojio, Yasutaka

Okada, Tsuyoshi

Okajima, Yoshiro

Okamoto, Yuri

Okazaki, Satoshi

Okino, Kazumaro

Okubo, Ryo

Okumura, Yasuyuki

Okuyama, Junko

Okuyama, Shigeki

Omiya, Yuki

Ono, Kazuya

Ono, Yuko

Orui, Masatsugu

Ostinelli, Edoardo G.

Ota, Miho

Ota, Toyosaku

Otaka, Yasushi

Otowa, Takeshi

Otsuka, Ikuo

Otsuka, Kotaro

Otsuki, Koji

Oya, Nozomu

Oya, Yoshio

Pichayapinyo, Panan

Pratim, Partha

Raballo, Andrea

Rayani, Milad

Rossetti, Ileana

Rossetto, Alyssia

Rukmani, Malligurki Raghurama

Sadahiro, Ryoichi

Sahker, Ethan

Saito, Junichi

Saito, Takeo

Saito, Taku

Sakamoto, Yui

Sakata, Masatsugu

Sakayori, Takeshi

Sakurai, Hitoshi

Salwan, Aaron

Sasabayashi, Daiki

Sato, Shinji

Schoretsanitis, Georgios

Seki, Masaki

Shibata, Mamoru

Shibata, Masahiko

Shida, Junko

Shigemura, Jun

Shimizu, Eiji

Shimizu, Isao

Shimizu, Kanako

Shimizu, Toshiyuki

Shimoda, Kengo

Shimode, Takaki

Shiozaki, Kazumasa

Shiratori, Yuki

So, Ryuhei

Sone, Daichi

Spina, Edoardo

Storck, Timo

Sugibayashi, Minoru

Sugiyama, Takehiro

Sumiyoshi, Tomiki

Suwa, Taro

Suzuki, Keisuke

Suzuki, Masahiro

Tachikawa, Hirokazu

Tada, Mariko

Tada, Mitsuhiro

Tajika, Aran

Takagi, Shunsuke

Takahashi, Michio

Takahashi, Nagahide

Takahashi, Shun

Takahashi, Tomohisa

Takano, Harumasa

Takekita, Yoshiteru

Takeshima, Masahiro

Takeshima, Tadashi

Takeuchi, Hiroyoshi

Takeuchi, Takashi

Tamada, Yu

Tamura, Takehiro

Tanaka, Kiwamu

Tanaka, Masuo

Tani, Hideaki

Tanimukai, Hitoshi

Tateno, Amane

Tateno, Ayumu

Tateno, Masaru

Terayama, Takero

Terui‐Honma, Toshihiro

Tochigi, Mamoru

Toda, Hiroaki

Toda, Hiroyuki

Togasaki, Yasuko

Tokumitsu, Keita

Tolcha, Abayneh

Tomioka, Hiroi

Tomita, Tetsu

Tomiyama, Hirofumi

Toro, Pablo

Toyomaki, Atsuhito

Trevino, Nancy

Tsoh, Janice

Tsuboi, Takashi

Tsuchiya, Kenji

Tsujii, Noa

Tsukuda, Banri

Tzeng, Nian‐Sheng

Uchida, Hiroyuki

Uchida, Takahito

Uchino, Takashi

Uchinuma, Niina

Ueda, Keita

Ueno, Senkei

Umegaki, Yusuke

Usami, Masahide

Usuda, Kentaro

Uwatoko, Teruhisa

Wada, Ken

Wada, Masataka

Wada, Yoshihisa

Watanabe, Shintaro

Waters, Kristin

Yada, Yuji

Yamada, Atsurou

Yamada, Hisashi

Yamada, Kazuo

Yamada, Shinichi

Yamada, Yuto

Yamagata, Hirotaka

Yamaguchi, Junji

Yamaguchi, Sosei

Yamamoto, Akira

Yamamoto, Kenji

Yamamoto, Naoki

Yamamuro, Kazuhiko

Yamanashi, Takehiko

Yamashita, Hiroshi

Yano, Shungo

Yasuda, Kazuyuki

Yasuda, Yuka

Yasumi, Katsuhiro

Yatomi, Taisuke

Yildiz, Esra

Yokode, Akiyoshi

Yokota, Osamu

Yoneoka, Daisuke

Yoshida, Kazunari

Yoshida, Mari

Yoshikawa, Eisho

Yoshimura, Masafumi

Yoshimura, Reiji

Yoshinaga, Naoki

Young, Allan

